# Monitoring Water Absorption and Desorption in Untreated and Consolidated Tuff by a Non-Invasive Graphene-Based Humidity Sensor

**DOI:** 10.3390/ma16051878

**Published:** 2023-02-24

**Authors:** Federico Olivieri, Rachele Castaldo, Gennaro Gentile, Marino Lavorgna

**Affiliations:** 1 Institute of Polymers, Composites and Biomaterials, National Research Council of Italy, Via Campi Flegrei 34, 80078 Pozzuoli, Italy; 2 Institute of Polymers, Composites and Biomaterials, National Research Council of Italy, P.le E. Fermi 1, 80055 Portici, Italy

**Keywords:** stone, conservation, water absorption, water desorption, humidity, graphene sensor

## Abstract

A hybrid montmorillonite (MMT)/reduced graphene oxide (rGO) film was realised and used as a non-invasive sensor for the monitoring of water absorption and desorption in pristine and consolidated tuff stones. This film was obtained by casting from a water dispersion containing graphene oxide (GO), montmorillonite and ascorbic acid; then the GO component was thermo-chemically reduced and the ascorbic acid phase was removed by washing. The hybrid film showed electrical surface conductivity that varied linearly with the relative humidity, ranging from 2.3 × 10^−3^ S in dry conditions to 5.0 × 10^−3^ S at 100% RH. The sensor was applied onto tuff stone samples through the use of a high amorphous polyvinyl alcohol layer (HAVOH) adhesive, which guaranteed good water diffusion from the stone to the film and was tested during water capillary absorption and drying tests. Results show that the sensor is able to monitor water content changes in the stone, being potentially useful to evaluate the water absorption and desorption behaviour of porous samples both in laboratory environments and in situ.

## 1. Introduction

Natural stone is a sustainable building material with an intrinsically low carbon footprint [1] that has high relevance in cultural heritage since mankind has always explored and exploited stone throughout history for different purposes [2]. Preserving and restoring stone-built heritage is, thus, one of the more relevant topics of conservation and, for this reason, the understanding of stone decay phenomena has been widely investigated in the past [3]. Stone decay can occur through different mechanisms, including the chemical action of anthropic pollutants, the physico-chemical degradation, induced by water-soluble salt migration and crystallisation, and the biodegradation [4]. Moreover, freezing–thaw cycles can quickly promote the degradation of stones, especially in the presence of a high water content [5].

Thus, water plays a crucial role in stone degradation. Water can induce degradation of stone by promoting a loss of cohesion of its structure, mechanical stresses as a result of the recrystallisation of soluble salts, and/or freezing–thaw cycles or chemical corrosion by an uptake of water-dissolved pollutants [6]. Moreover, the kinetic and the extent of stone biodegradation phenomena are strongly influenced by water availability [7]. Water is, therefore, commonly regarded as the main factor affecting the degradation mechanisms of stones, acting as a primary degradation agent through freeze–thaw cycles or the dissolution of the stone binders, by acting as a solvent and transport medium for other degradation agents, such as pollutants or water-soluble salts, or by creating high humidity conditions necessary for biological degradation.

For these reasons, the investigation of stone decay phenomena has always been associated with the study of water content in the porous structure of stone. Monitoring water absorption and desorption phenomena is thus a very relevant subject to evaluating the susceptibility of stone structures to degradation. Moreover, the analysis of the water absorption/desorption behaviour is always performed to evaluate the efficiency of protective and consolidating treatments on stone and the preserved breathability of the substrate after the application of the treatments [8,9,10].

For the evaluation of the water content in stones, in addition to classical gravimetric analysis [11], which is easily performed in laboratory but unsuitable for in-situ applications, different methods have been developed in past years. Time domain reflectometry (TDR) [12,13], frequency domain reflectometry (FDR) [14], mercury intrusion porosimetry and nitrogen adsorption techniques [15] have been tested. Further techniques have been developed to evaluate water content, specifically in stone cultural heritage artworks. Nuclear magnetic resonance (NMR) as well as magnetic resonance imaging (MRI), tuned to excite the hydrogen nucleus, have been widely applied because they are particularly sensitive to water molecules [6,16,17]. Near-infrared spectroscopy (NIRS) [18], IR thermography [19,20], ultrasound pulse velocity (UPV) [21], evanescent field dielectrometry (EFD) [22], scattering of neutrons [23], microwave and radar [24] have been also applied. Optical fiber-based technologies, such as distributed temperature sensing (DTS) [25] and fiber Bragg grating (FBG) [26], represent excellent sensors to detect the water content of stone.

Nevertheless, all these techniques are based on complex analytical techniques and most of them are invasive and difficult to apply in situ for cultural heritage purposes. This represents a severe limitation since the morphology and the structure of the artifacts must be unaffected and a versatile approach to testing artworks of different sizes is often required [27]. Therefore, several researchers are currently focused on the development of sensors able to overcome in-situ application limitations due to technical problems or low resolution [25,28,29]. The latter problem is particularly important, especially during the drying process of stones, because, in general, water content measurement techniques are more precise during the capillary rise, being less well-performing when the water content decreases [30].

In a previous work, bio-inspired and sustainable self-standing hybrid films with good mechanical properties were obtained by casting graphene oxide (GO)/montmorillonite water dispersions, exhibiting high sensitivity to humidity [31]. After a mild thermal reduction of the GO phase, the films showed electrical conductivity variable with the relative humidity, due to their water absorption/desorption capability. At high water contents, their electrical conductivity was enhanced due to the improved ion conductivity of the montmorillonite phase. Nevertheless, for graphene-based hybrids, it has been reported that thermal reduction at moderate temperatures often leaves high amounts of residual oxygen-containing groups and structural defects that preclude the obtainment of high electrical conductivity [32,33]. As an alternative strategy to improve the electrical response of graphene materials and related hybrids, different thermo-chemical reduction processes of the GO phase are proposed. In particular, an effective and sustainable mild thermo-chemical reduction of GO is based on the use of a green chemical reduction agent, namely ascorbic acid [34,35]. Additionally, thermo-chemically reduced GO coatings can show variable responses to relative humidity as water molecules intercalate in the graphene structures, causing the enhancement of the proton conductivity of graphene-based materials [36,37].

On the basis of exploiting the variable electrical conductivity of reduced graphene oxide (rGO)/montmorillonite hybrids when exposed to environments with variable water vapour contents, this work was focused on the development of a non-invasive novel sensor that is able to monitor water absorption and desorption in pristine and consolidated porous substrates. The working principle at the basis of the sensor is that, when placed in contact with a porous substrate with variable water content—due, for instance, to water absorption/desorption phenomena—the rGO/montmorillonite hybrid film is exposed to variable water vapour flows, thus showing an electrical conductivity that changes during the process. Thus, the electrical conductivity changes can be potentially correlated to the water content changes occurring in the porous structures. The main potential advantages of such a sensor are its non-invasiveness, as the sensor can be installed on the surface of a porous stone substrate without the necessity of introducing the sensing elements inside the material, its ability to provide readouts without changing the structure of the material, its easy removing [38] and its ability to monitor water changes in real time. 

Exploiting this concept, a hybrid rGO/montmorillonite film was obtained by water casting with mixed GO/montmorillonite/ascorbic acid water dispersions and by thermo-chemical reduction at mild temperatures. The use of ascorbic acid as an environmentally sustainable reducing agent was foreseen to enhance and tailor the electrical conductivity of the hybrid rGO/montmorillonite film. After ascorbic acid removal, the electrical surface conductivity of the film was tested at variable RH. Then, the film was tested as a sensor to evaluate the water content variation inside a stone substrate. Tuff, a high porous stone with high water absorption capability, was selected as a substrate. This is very relevant to cultural heritage applications due to the large number of ancient tuff monuments, sculptures and artefacts located in countries rich of volcanic deposits, such as Italy, Turkey, Mexico, Germany and Japan [39]. Moreover, due to its pronounced tendency to degradation through different mechanisms, tuff often needs periodical conservation treatments. Several consolidation approaches have been proposed in the past to reduce the inner tuff porosity and improve the hydrophobicity of the stone [40,41,42,43]; in order to check the effectiveness of these conservation treatments, approaches able to evaluate the water content in tuff’s porous structure are very useful.

To improve the contact with the stone and its water monitoring ability, the film was applied to the tuff surface through a water-sensitive adhesive layer constituted by high amorphous polyvinyl alcohol (HAVOH), a highly interesting polymer due to its excellent processability, water solubility and ease for coating [44]. The electrical response of the sensor was then tested during water capillary absorption and drying tests and compared with the water content evaluation performed by gravimetrical analysis. The results indicated that the developed sensor is highly promising for the non-invasive and real-time monitoring of water content in porous substrates. Indeed, it is able to well detect when water absorbed by capillarity reaches the surface of the stone where the sensor is placed, showing a steep increase of its electrical response, and it is able to real-time monitor the drying behaviour of the stone, showing a progressive decrease of its electrical conductivity. 

## 2. Materials and Methods

### 2.1. Materials

Sodium intercalated montmorillonite (MMT) was purchased from BYK-Chemie GmbH (Wesel, Germany). Graphene oxide water dispersion (4.0 g L^−1^) was purchased from Nanesa S.r.l (Arezzo, Italy). Ascorbic acid (AA) was purchased from Sigma-Aldrich (St. Louis, MO, USA). HAVOH (commercial name G-Polymer) was obtained by Mitsubishi Chemical Corporation (Tokyo, Japan). The water-repellent organic-modified silane and siloxane-based dispersion Achibuild CB ECO (Achibuild, dry content 60 wt%), which is a commercial consolidation product for tuff, was received from Achitex Minerva (Vaiano Cremasco, Italy). Neapolitan tuff was obtained from a local cave and had density of 1.2 ± 0.3 cm^3^ and a porosity of 65.9 ± 1.7%. Tuff samples had dimensions of 5 × 5 × 2 cm^3^. Specimens were washed with distilled water and dried until constant weight before the tests.

### 2.2. Sample Impregnation and Evaluation of the Amount of Consolidating Agent Applied

First, 15 g of Achibuild were diluted with distilled water to 10% dry content. The dispersion was then applied on the tuff specimen’s surface by pipette until saturation [45]. The treated specimen was coded as TUFF-C. Before the treatment, the tuff specimen was dried at 60 °C under vacuum for 1 week and thereafter conditioned at 25 °C and 50% RH for 1 week until constant weight. 15 days after the treatment, the specimens were subjected to the same conditioning cycle. After conditioning, in order to determine the amount of material adsorbed, the weight of each specimen was recorded again. The amount of dried, adsorbed material per specimen was measured by gravimetric analysis and resulted 1.88 wt%, with respect to the dried weight of the untreated tuff specimen. Considering the dimensions of the treated surface, this value corresponds to an amount of applied material per surface area of 584 g/m^2^.

### 2.3. Film Preparation

An MMT/rGO film was prepared as follows. MMT was dispersed in distilled water at a 4.0 g L^−1^ concentration by ultrasonication using a Sonics Vibracell ultrasonic processor (500 W, 20 kHz) at 25% of amplitude for 30 min, with 30 s/30 s on/off cycles. Subsequently, the MMT and commercial GO dispersions were mixed to obtain an MMT/GO dispersion with the MMT:GO weight ratio as 4:1. A 200 wt% of AA with respect to the dry weight of GO was added to the mixture. This dispersion was ultrasonicated for 15 min with 30 s/30 s on/off cycles and then poured in a petri dish to obtain an MMT/GO/AA film with 20 µm thickness upon water evaporation. The Petri dish was kept on a levelled laboratory bench at room temperature until complete drying, which occurred in about 60 h. The thermo-chemical reduction of the MMT/GO/AA film was promoted by heating the film at 180 °C in an oven for 2 h. Then, AA was removed by repeated washings with distilled water, thereby obtaining the MMT/rGO film.

### 2.4. Sensor Application

**Figure 1:**
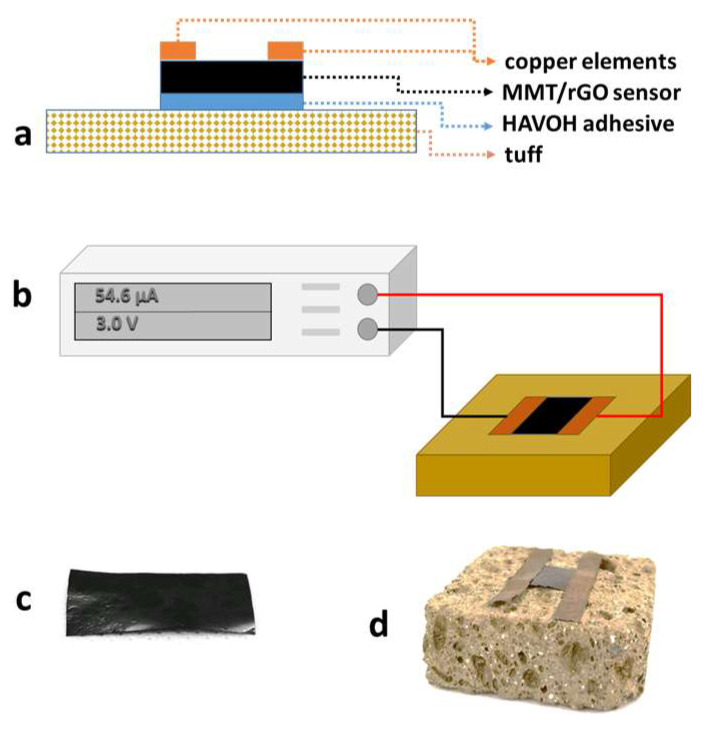
(**a**) Scheme of the MMT/rGO sensor applied onto the tuff specimen through the HAVOH adhesive layer; (**b**) scheme of experimental set-up used to evaluate the electrical response of the MMT/rGO sensor on the tuff specimens during the water capillary absorption test and the drying index test; (**c**) image of the MMT/rGO film; (**d**) image of the MMT/rGO sensor installed on a tuff specimen.

A 20 wt% aqueous solution of HAVOH was used to apply an adhesive layer between the tuff stone and the MMT/rGO film (Figure 1a). Specifically, HAVOH was dissolved in water at 80 °C. After the cooling of the solution, 50 mg of HAVOH solution were applied onto a 1 cm^2^ area of the untreated and consolidated TUFF and TUFF-C samples. Then, MMT/rGO films with the same area were placed in contact with the HAVOH layer and the HAVOH was left to dry in laboratory conditions.

### 2.5. Morphological Characterisation

The MMT/rGO surface and cross-section were observed by Scanning Electron Microscopy (SEM), using a FEI Quanta 200 FEG (FEI, Eindhoven, The Netherlands) in high vacuum mode, at 10–20 kV acceleration voltage using a secondary electron detector (FEI, Eindhoven, The Netherlands). Before SEM observations, the film was mounted onto SEM stubs by means of carbon adhesive disks. Energy Dispersive X-ray (EDX) local analysis and mapping were also performed on the film surface and cross-section, respectively, by using the same instrument equipped with an Oxford Inca Energy System 250 and an Inca-X-act LN2-free analytical silicon drift detector (FEI, Eindhoven, The Netherlands).

### 2.6. Capillary Absorption and Desorption Tests

Water capillary absorption tests were carried out on untreated TUFF in accordance with the standard EN 15801:2009 [46]. The specimens were placed on a filter paper pad soaked in deionised water, with the treated surface in contact with the pad. The amount of absorbed water, Ai, at time, ti, per surface unit was calculated according to the following equation:

A_i_ = (m_i_ − m_dry_)/S (1)

where m_dry_ is the weight of the dry specimen, m_i_ is the weight of the specimen after absorbing water for a period of time, t_i_, and S is the surface of the specimen kept in contact with water. Water capillary absorption curves were obtained by plotting A_i_ (g cm^−2^) versus time^1/2^ (s^1/2^). The capillary water absorption coefficient (AC), (g cm^−2^ s^−1/2^) was obtained from the initial slope of the curve.

Drying index tests were carried out on untreated and treated TUFF and TUFF-C specimens in accordance with the standard EN 16,322:2013 [47]. Previously dried specimens were immersed in distilled water until constant weight (about 1 week). Thereafter, specimens were placed in a climatic chamber and left to dry at 25.0 ± 0.1 °C and 50 ± 2% RH, while their mass was monitored to determine the loss of water. 

The residual water content in the stone at the time, i, was calculated as:

W_j_ = (m_j_ − m_dry_)/S (2)

where m_dry_ is the weight of the dry specimen, m_j_ is the weight of the specimen at the time, j, from the beginning of the drying test, and S is the surface of the specimen kept in contact with water. The drying curve was obtained by plotting W_j_ (g cm^−2^) versus time (min). The drying behaviour of each specimen was evaluated through the determination of the drying rate corresponding to the first drying phase (D/W_0_), which is the negative slope of the initial linear part of the drying curve, obtained by plotting the residual amount of water present in the specimen per unit area against time (min), normalised by the amount of water absorbed by the sample at the beginning of the test (W_0_).

### 2.7. Electrical Characterisation

All the electrical characterisations were performed by means of a Keithley 2450 SourceMeter multimeter (Cleveland, OH, USA) with two probes, by applying a 3 V voltage.

Surface conductivity of MMT/rGO and MMT/GO/AA were elaborate by applying the equation:

σ_S_ = (I L)/(V d) (3)

where σ_S_ is the surface conductivity, I is the current, V is the applied voltage, d is the length of the sensor (i.e., the distance between the electrodes), and L is the length of the sensor along its cross section (in these tests, d and L were 1 cm). Tests were registered in triplicate; average results are reported. The surface conductivity was also evaluated by progressively increasing the humidity of the environment from 10% to 90% RH and by the sudden increase of the humidity from 10% to 100% RH.

The electrical tests on the MMT/rGO sensor were performed by connecting the multimeter to the sensor applied on the stones through copper adhesive tapes; the scheme of the setup is showed in Figure 1b. All the tests were conducted at room temperature, with relative humidity of 50% and by imposing a 3 V voltage between the electrodes. The current through the sensor was measured during water capillary absorption tests performed on the untreated TUFF specimen and during drying index tests performed on TUFF and TUFF-C specimens. Results were reported by calculating the surface conductivity, applying the Equation (3).

## 3. Results

### 3.1. MMT/rGO Film Characterszation

**Figure 2:**
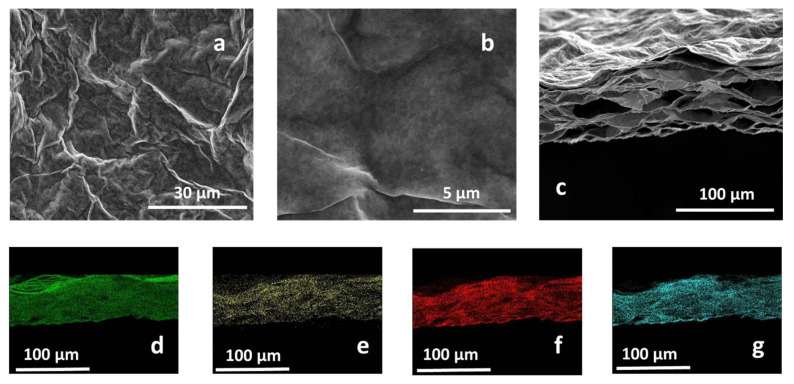
Secondary electron SEM images of the MMT/rGO film surface (**a**,**b**) and cross-section (**c**). EDX mapping of the film cross-section, evidencing the homogeneous distribution of Al (**d**), C (**e**), O (**f**), and Si (**g**).

MMT/rGO thin films were obtained through self-assembly at the liquid–air interface of montmorillonite/graphene oxide hybrids in the presence of ascorbic acid and through the thermo-chemical reduction of GO. After the ascorbic acid removal, SEM analysis of MMT/rGO revealed a highly wrinkled surface (Figure 2a,b) of the film and the formation of a stable hybrid network, characterised by diffuse porosities, elongated in the longitudinal direction of the film, to be ascribed to the thermo-chemical reduction process of the rGO phase (Figure 2c). EDX mapping performed on the cross-section of the film (Figure 2d–g) showed the compositional homogeneity of the hybrid film, as proven by the similar distribution of aluminium (Figure 2d), carbon (Figure 2e), oxygen (Figure 2f) and silicon (Figure 2g) through its section. Moreover, EDX analysis performed on an rGO sample obtained by thermo-chemical reduction in the presence of ascorbic acid revealed a C/O atomic ratio of (5.24 ± 0.19), significantly higher than the C/O atomic ratio found on thermally reduced rGO samples (2.91 ± 0.12) [31], confirming the effectiveness of ascorbic acid in enhancing the reduction extent of the GO phase.

**Figure 3:**
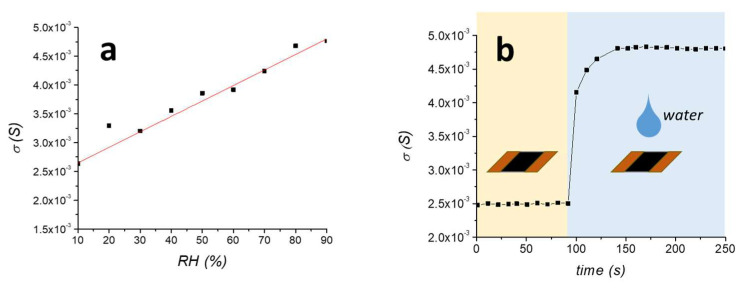
Surface conductivity of MMT/rGO film by progressively increasing the relative humidity (**a**) and by quick wetting of the dried film (**b**).

The surface conductivity of MMT/rGO, measured at 25°C and 50% RH, was (3.75 ± 0.26) × 10^−3^ S, which is more than two orders of magnitude higher than the non-reduced film MMT/GO/AA (8.83 ± 0.64) × 10^−6^ S. Moreover, the surface conductivity of the film varied with the relative humidity, as evident in Figure 3. In particular, the σ_S_ increasing is almost linear with RH (R^2^ = 0.964), ranging from 2.7 × 10^−3^ S at 10% RH to 4.7 × 10^−3^ S at 90% RH (Figure 3a). This result is different than what was previously obtained on thermally reduced MMT/rGO films, which showed an electrical surface conductivity variable with the increase of RH with an exponential trend [31]. This difference can be explained by the lower hydrophilicity of the film obtained by thermo-chemical reduction due to the improved reduction extent of the GO phase, as shown by the higher C/O ratio evidenced by EDX, in comparison with the thermally reduced rGO sample. Moreover, the electrical response of the film was evaluated by completely drying the film and then applying a water drop to its surface. As shown in Figure 3b, the increase of σ_S_ was instantaneous and the surface conductivity resulted as stabilised in about 40 s after the drop application.

### 3.2. Water Capillary Absorption

**Figure 4:**
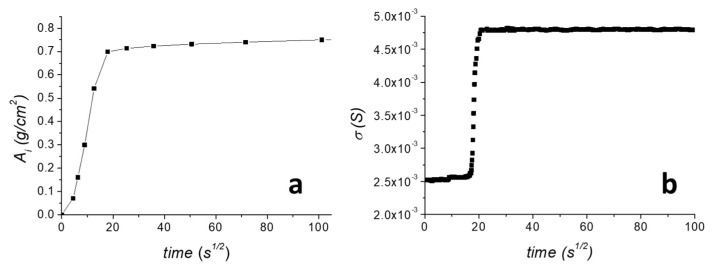
(**a**) Water capillary absorption curve of untreated TUFF specimen. (**b**) Surface conductivity profile recorded at the sides of the MMT/rGO sensor applying a 3 V voltage during the water capillary absorption test.

To evaluate the electrical response of the graphene sensor during water capillary absorption, a water capillary absorption test of the untreated TUFF was performed. Results are reported in Figure 4. As shown in Figure 4a, the amount of absorbed water, Ai, progressively increased during the first 18 s^1/2^ of the test, and then slowly reached a plateau, corresponding to the maximum amount of water that could be absorbed by the specimen during the remaining time. The capillary water absorption coefficient, AC, was found 20.2 g cm^−2^ s^−1/2^. After drying the sample, the same experiment was performed by mounting the sensor on the sample surface, as previously reported in Figure 1, and recording the current flowing through the graphene film by application of a 3 V voltage. The surface conductivity plot is reported in Figure 4b. In order to compare the surface conductivity plot with the water capillary absorption curve, σ_S_ was plotted vs. the square root of the time (s^1/2^). As shown, in contrast to what was observed for the amount of absorbed water, σ_S_ was almost constant in the first 20 s^1/2^ of the experiment and showed a very steep increase only in the correspondence of the plateau value reached by the absorbed water. This can be well explained considering that capillary water absorption is a fast phenomenon and during the test the water distribution in the specimen was highly inhomogeneous. Nevertheless, the sensor was well able to detect the time when water reached the upper surface, where the sensor was placed, as only at this point water was absorbed by the sensor element and induced a rapid increase of σ_S_. To better evidence the different behaviour of the absorbed water curve and the surface conductivity curve during the water capillary absorption test, both Ai and σ_S_ are plotted vs. the square root of time in the first part of the curve (Figure S1). It is well evidenced that the sensor, failing to follow the progressive increase of the water absorbed into the stone, is in any case effectively able to provide a clear on/off response in correspondence with the point when water reaches the upper surface of the specimen, and thus, it starts to be absorbed by the sensor element itself.

### 3.3. Drying Index

The efficiency of the MMT/rGO sensor was then tested by the evaluation of the drying index of the tuff stones. For this test, as detailed in the experiment, an untreated sample (TUFF) as well as a sample treated with a commercial consolidating agent (TUFF-C) were tested.

**Figure 5:**
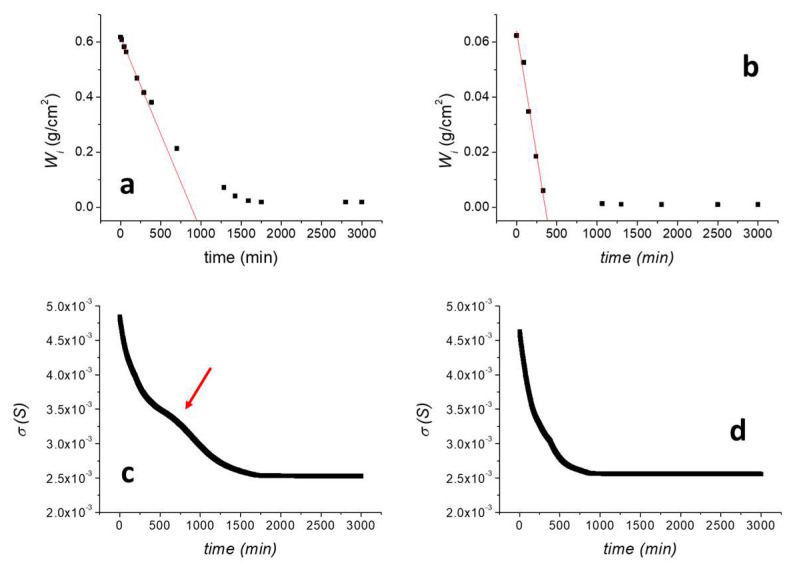
(**a**) Drying curve of the untreated TUFF sample; (**b**) drying curve of the consolidated TUFF-C sample; (**c**) surface conductivity of the sensor applied on the untreated TUFF sample during the drying test; (**d**) surface conductivity of the sensor applied on the consolidated TUFF-C sample during the drying test.

The drying rate was preliminarily evaluated as measuring D/W_0_ (min^−1^), defined as the negative slope of the linear part of the drying curve obtained by plotting the residual amount of water present in the specimen per unit area against time (min), normalised to the amount of water in the sample at the beginning of the test (W_0_). Results are reported in Figure 5a for the untreated TUFF sample and in Figure 5b for the consolidated TUFF-C sample. As shown, the consolidating treatment induced a significant reduction of the maximum amount of absorbed water. Indeed, while the maximum water absorption for TUFF is comparable to the maximum value obtained by the capillary absorption test, for the TUFF-C sample the maximum amount of absorbed water decreased about one order of magnitude. The drying rate was not negatively affected by the treatment. Indeed, D/W_0_ increased from (1.13 ± 0.21) × 10^−3^ min^−1^ for TUFF (R^2^ = 0.998) to (2.88 ± 0.30) × 10^−3^ min^−1^ for TUFF-C (R^2^ = 0.977), confirming that the treatment still ensures a good breathability to the consolidated stone.

The surface conductivity of the sensor applied onto TUFF (Figure 5c) showed a progressive decrease from 4.81 × 10^−3^ S to about 2.68 × 10^−3^ S at about 1600 min when it reached the current plateau value, well matching the water desorption curve shown in Figure 5a. Nevertheless, a clearly visible change in the slope was observed between 500 and 1000 min (indicated with a red arrow in Figure 5c), not associated with any water desorption phenomenon. This can be explained by considering that the adhesive HAVOH layer partially retained part of the high amount of quickly desorbed water from the tuff sample during the first part of the test, inducing a deviation of the surface conductivity response in comparison with those predictable by the kinetic of water desorption from the stone. To confirm this behaviour, a desorption test on the TUFF sample was performed by doubling the thickness of the HAVOH adhesive layer (Figure S2). In this case, the slope variation between 500 and 1000 min is much more evident, confirming that it is related to the liquid water fraction absorbed by the adhesive and diffusing to the sensor. After that time, the water content in the adhesive layer equilibrates with the water content in the stone and the surface conductivity curve returns to well follow the water desorption trend.

Concerning the consolidated TUFF-C sample, the surface conductivity of the sensor during the drying process (Figure 5d) showed a progressive decrease from about 4.67 × 10^−3^ S to 2.62 × 10^−3^ S in the first 750 min of the test and then reached a plateau. This behaviour is very similar to the water desorption curve of the TUFF-C sample (Figure 5b), as for this sample most of drying process occurred in the first 750 min of the experiment, when the residual amount of water in the sample reached a plateau value. Thus, for this sample, there is a better correlation between the sensor response and the residual amount of water in the tuff sample during drying.

## 4. Concluding Remarks

A thin and flexible MMT/rGO hybrid film with variable electrical conductivity when exposed to environments with variable humidity was exploited as a novel, non-invasive sensor to monitor water content variations in tuff stone. The hybrid film was obtained by self-assembly of GO and MMT and through a mild, environmentally friendly thermo-chemical reduction of GO induced by ascorbic acid. After ascorbic acid removal, the MMT/rGO hybrid film showed a homogenous structure and an electrical surface conductivity dependent on environmental moisture (2.7 × 10^−3^ S at 10% RH to 4.7 × 10^−3^ S at 90% RH), which is very interesting for humidity-sensing applications. The film was then tested on tuff samples during capillary water absorption and drying index tests. To ensure a good contact with the stone, the MMT/rGO film was engineered through the application of a HAVOH adhesive layer that was able to ensure the diffusion of the humidity from the stone to the sensor. The obtained results indicate that the developed sensor is highly promising for the non-invasive and real-time monitoring of water content in porous substrates. Concerning the capillary water absorption behaviour, the sensor was able to give a very quick response when water absorbed by capillarity reached the upper surface of the specimen, showing a steep conductivity increase associated with the occurrence of direct contact of liquid water with the sensor. As concerns the drying process, the sensor showed a progressive decrease of its electrical conductivity, with the electrical conductivity curve well matching the drying behaviour. For the untreated tuff sample, during the drying test, the surface conductivity of the sensor applied to the tuff’s surface showed a progressive decrease from 4.81 × 10^−3^ S to about 2.68 × 10^−3^ S at about 1600 min, when it reached the current plateau value. The result was similar for a consolidated tuff sample. During drying, the surface conductivity of the sensor showed a progressive decrease from about 4.67 × 10^−3^ S to 2.62 × 10^−3^ S in the first 750 min of the test and then reached a plateau. The surface conductivity curves well matched the water desorption curve, indicating the possible applicability of the proposed sensor for the real-time monitoring of changes in the amount of water in tuff. 

The ability of the sensor to evaluate water absorption and desorption behaviour of tuff in real time and its possible usefulness for monitoring these processes also in situ are very relevant, as the sensor can provide useful information to predict degradation phenomena associated with the effect of water and to plan interventions to counter them. Moreover, the sensor can be useful to evaluate the effect of consolidation treatments on tuff artifacts and structures, giving information on the effect of the treatment on the breathability of the treated substrates.

Although further activities are needed to validate the sensor on different porous substrates, the very simple working principle of the sensor can allow its exploitation for the monitoring of water absorption/desorption also on different stones of historical and cultural interest, as well as on new construction materials. 

## Data Availability

The data presented in this study are available on request from the corresponding author.

## Supplementary Materials

The following supporting information can be downloaded at https://www.mdpi.com/article/10.3390/ma16051878/s1, Figure S1: Overlapped plot of the amount of absorbed water, Ai, and the surface conductivity in the first part of the experiment; Figure S2: Surface conductivity of the sensor, applied with a thicker HAVOH adhesive layer on the untreated TUFF sample, during the drying test.
