# Supplementary material for: Monitoring Water Absorption and Desorption in Untreated and Consolidated Tuff by a Non-Invasive Graphene-Based Humidity Sensor
Source: Materials (Basel). 2023 Feb 24;16(5):1878. doi: 10.3390/ma16051878 (PMC10004346; doi:10.3390/ma16051878)
Supplement: Supplementary file 1 [file materials-16-01878-s001.zip › materials-2229427-supplementary.pdf]

Supplementary Information

Monitoring Water Absorption and Desorption in Untreated and Consolidated Tuff by a Non-Invasive Graphene-Based Humidity Sensor

Federico Olivieri [1], Rachele Castaldo [1], Gennaro Gentile [1,*] and Marino Lavorgna [2]

[1] Institute of Polymers, Composites and Biomaterials, National Research Council of Italy, Via Campi Flegrei 34, 80078 Pozzuoli, Italy

[2] Institute of Polymers, Composites and Biomaterials, National Research Council of Italy, P.le E. Fermi 1, 80055 Portici, Italy

* Correspondence: gennaro.gentile@cnr.it

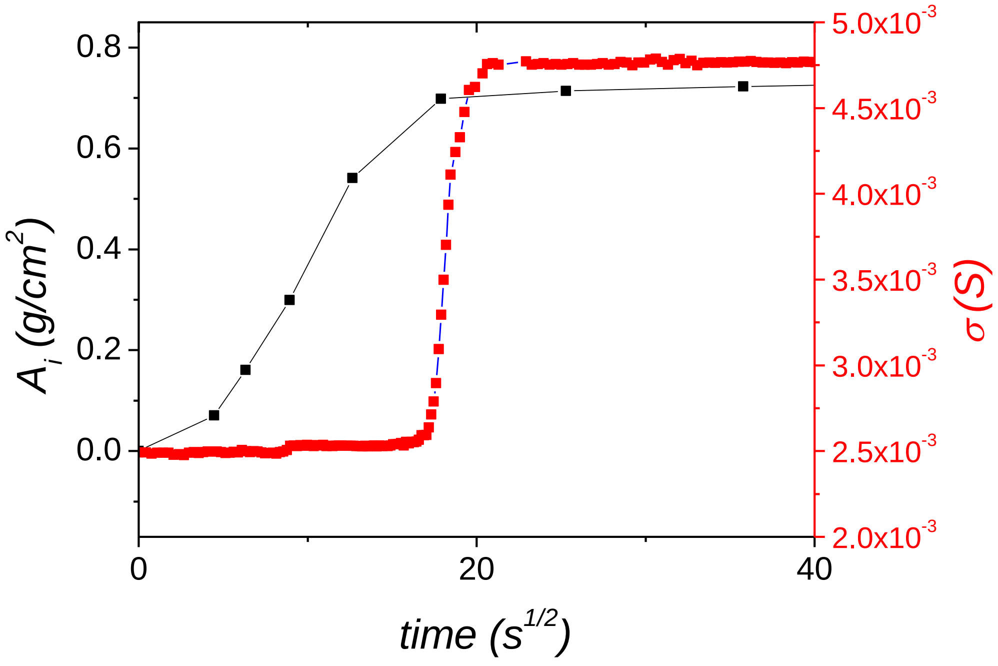

Figure S1. Overlapped plot of the amount of absorbed water A_i and the surface conductivity in the first part of the experiment.

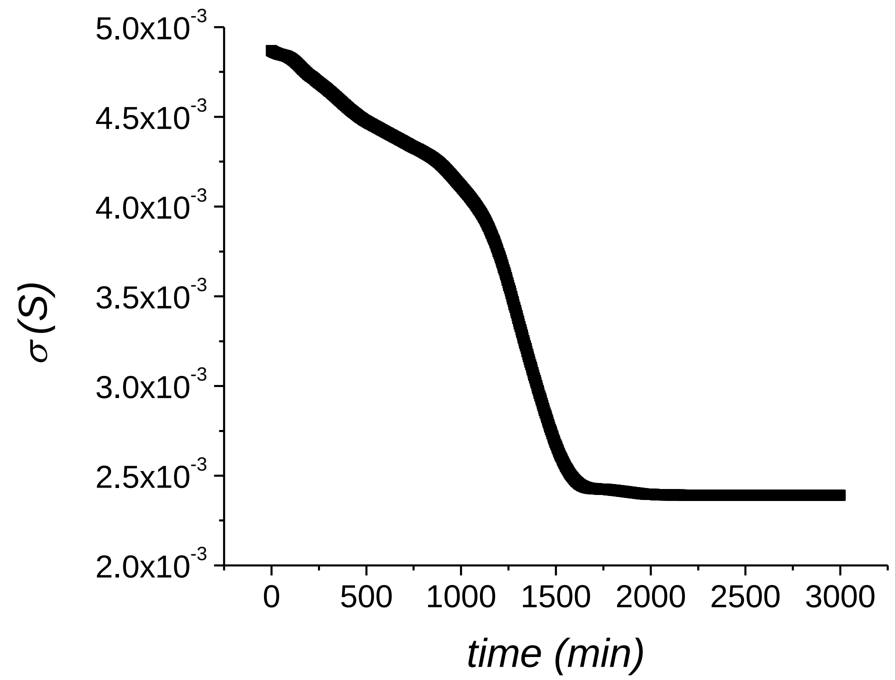

Figure S2. Surface conductivity of the sensor, applied with a thicker HAVOH adhesive layer on the untreated TUFF sample, during the drying test.
